# Evolution of Clinical Care in COVID-Infected Solid Organ Transplant Recipients

**DOI:** 10.1007/s40472-022-00368-z

**Published:** 2022-05-31

**Authors:** Rachel Sigler, Victor Chen, Nancy Law

**Affiliations:** 1 Division of Infectious Diseases and Global Public Health, University of California San Diego, 9444 Medical Center Drive, MC 0879, La Jolla, CA 92093-0879 USA; 2 Department of Pharmacy, University of California San Diego, La Jolla, CA USA

**Keywords:** COVID-19, Solid organ transplant, Treatment

## Abstract

**Purpose of Review:**

In this review, we aim to summarize the evolution of care for the solid organ transplant recipient (SOTR) with COVID-19 disease, based on the current published guidelines and our center’s experience.

**Recent Findings:**

Oral antiviral medications and monoclonal antibodies are now used with the goal to prevent severe disease. Immunomodulating drugs in addition to antivirals have been used in the treatment of severe COVID-19.

**Summary:**

With the ongoing pandemic and unique challenges posed by the SOTR, understanding the risk and advancing management and treatment of COVID-19 infections are imperative to the successful care of a transplant recipient. There are many ongoing clinical trials being conducted in hopes of developing novel therapeutics towards COVID-19.

## Introduction

As the severe acute respiratory syndrome coronavirus 2 (SARS-CoV-2) pandemic is now entering its third year, treatment and prevention methods have evolved and multiplied. Numerous medications for the treatment of coronavirus disease 19 (COVID-19) have been studied, and some have received emergency use authorization (EUA) and Food and Drug Administration (FDA) approval, allowing for improved outcomes in the fight against this infection [1, 2•]. Solid organ transplant recipients (SOTRs) are at greater risk for severe COVID-19, with worse outcomes and higher mortality rates than immunocompetent patients [3]. In general, SOTRs were not specifically included in many of the large clinical trials of COVID-19 therapeutics, thus making associations in this population difficult. A majority of the evidence for the use of these novel therapeutics in SOTR comes from retrospective studies. It is crucial to perform more large-scale studies investigating COVID-19 treatment options in SOTR to improve outcomes in this population.

Prevention options for SOTR have become a priority to stop the spread of COVID-19 [4•]. With the advent of COVID-19 vaccinations in late 2020, multiple studies have attempted to evaluate their effectiveness. Demonstrating SOTRs’ inability to mount a strong antibody response to vaccination led to recommendations for a third dose [5••]. Unfortunately, breakthrough rates of COVID-19 after vaccination in SOTR are higher than in immunocompetent patients [6•]. In a large multicenter study of 18,215 fully vaccinated SOTR, breakthrough COVID-19 infections occurred in 0.23–2.52%, compared to 0.01% in the healthy vaccinated hosts [6•]. Therefore, investigations into medications as primary COVID-19 prevention are also important in SOTR, in addition to ongoing vaccination and booster studies.

This review will highlight the evolution of clinical developments in the treatment of COVID-19, particularly in infected transplant recipients. It will include discussions on prevention techniques and treatment options. Throughout this review, when discussing severity of COVID-19 disease, definitions will be consistent with the National Institutes of Health (NIH) COVID-19 Panel guidelines on the treatment of COVID-19 [7••]. It should be noted the information is based on the medical literature available as of February 15, 2022.

## COVID-19 Therapeutics

### Remdesivir

Early in the pandemic, remdesivir, an adenosine nucleotide pro-drug, was seen to be effective against COVID-19 [8]. In April 2020, the National Institutes of Health (NIH) released encouraging data based on the Adaptive COVID-19 Treatment Trial 1 (ACTT-1) on remdesivir. ACTT-1 revealed that among hospitalized patients with COVID-19 who required supplemental oxygen or ventilatory support, treatment with remdesivir decreased time to recovery compared to placebo [9••]. Early treatment was also better. The greatest benefit of remdesivir occurred when patients were randomized during the first 10 days after symptom onset [9••]. Likewise, in the DisCoVeRy randomized controlled trial (RCT), patients randomized to the remdesivir arm did not benefit from the treatment if they were symptomatic for more than 7 days [10••].

Treatment duration with remdesivir was investigated in two large open-label trials. The SIMPLE Trial randomized hospitalized patients on supplemental oxygen to either 5- or 10-day courses of remdesivir and found that a 5-day course of treatment was equally beneficial to a longer course by day 15 [2•, 7••, 11•]. In the second RCT, hospitalized patients with moderate COVID-19 were randomized to 5-day remdesivir, 10-day remdesivir, or standard of care; on day 11, patients in the 5-day arm had better outcomes than those receiving standard of care [12•]. The open-label nature of this trial allowed for a variety of concomitant medications used in all treatment arms, potentially affecting interpretation. However, 5-day treatment courses of remdesivir quickly became the drug regimen of choice to treat hospitalized patients with moderate COVID-19.

Based on preliminary early data, the FDA granted emergency use authorization (EUA) for remdesivir as of May 1, 2020 [13]. Furthermore, the FDA granted full approval for remdesivir for the treatment of moderate COVID-19 in hospitalized patients in October of 2020 with final data from the ACTT-1 and SIMPLE trials as well as other RCTs, as they showed decreased length of stay and reduced oxygen need [11•, 12•, 14]. Lately, the PINETREE study evaluated the use of remdesivir in mild to moderate COVID-19 treated in the outpatient setting [15••]. This study found that 3 days of remdesivir treatment reduced the risk of hospitalization or death by 87% compared to placebo among patients with mild COVID-19 symptoms for less than 7 days.[15••] Thus, as of January 21, 2022, the FDA expanded the EUA for remdesivir to include outpatient treatment for mild to moderate COVID-19 in patients over 12 years old [16].

It should be noted that benefits of remdesivir were not definitive in many trials, and no trial revealed a mortality benefit. In the ACTT-1 trial, patients who required high-flow oxygen, mechanical ventilation, or ECMO saw no difference in time to recovery.[9••] The Solidarity Trial by the World Health Organization (WHO) randomized hospitalized patients with COVID-19 of any severity to 5 arms, including 5 or 10 days of remdesivir and placebo. Compared to their control group, remdesivir did not reduce in-hospital mortality, the need for mechanical ventilation, or duration of hospitalization [17•]. This study was open-label and may have limited the ability to assess time to recovery. It was not cited in the FDA’s approval of remdesivir. The study did not include data on time from symptom onset to enrollment [7••]. In these trials, remdesivir appears to be most effective in hospitalized patients with moderate COVID-19 on supplemental oxygen [9••, 10••, 17•].

SOTRs were not explicitly excluded from the major trials of remdesivir. While transplant medications may have represented a contraindication to treatment arms, SOTR enrollment in these trials is not explicitly stated. The ACTT-1 trial did not specify how many SOTR were included; however, it is noted that of the 1062 participants, 6.9% had some type of immune deficiency, either acquired or innate [9••]. Studies for the use of remdesivir in SOTR include retrospective case series, most of which were conducted earlier in the pandemic [18, 19]. As remdesivir was still investigational at the time, these studies had low numbers of recruited patients and utilized additional medications that likely led to confounding [20].

One early retrospective cohort of SOTR include 90 patients, but remdesivir was utilized in only two patients, one with severe disease and one with moderate disease [21]. This initial survey of SOTR with COVID-19 demonstrated that SOTR had more severe disease than immunocompetent counterparts, making comparison of treatment outcomes difficult. Evidence for use of remdesivir in SOTR in published trials is limited, with the total number of patients less than 50, and study design limited by available patients [18, 19, 22–25]. The large randomized controlled trials (RCTs) conducted on remdesivir did not include sub-group analysis of transplant recipients or, in some cases such as the Solidarity Trial, did not include immunocompromised patients at all.

However, now that remdesivir has been extensively used in this pandemic, practitioners have developed an anecdotal body of evidence to comfortably implement its frequent use in SOTR. Prior to extensive use of remdesivir, early caution for liver and kidney donors was advised due to remdesivir adverse effects. Indeed, in Wang et al. as well as in the ACTT-1 trial, elevations in transaminases more than five times the upper limit of normal as well as renal impairment with glomerular filtration rate (GFR < 30 mL/min) or renal replacement therapy were exclusion criteria for the trials [9••, 26]. Further case–control studies have found that remdesivir is safe to use, even in patients with impaired renal function [27]. See Table 1 for authorization status, mechanism of action, administration guidelines, and important interactions for remdesivir.

**Table 1:** COVID-19 therapeutics for SOTR

Medication	COVID-19 FDA authorization status*	Mechanism of action	Variant activity	Indication/dosage administration	Risks/contraindications	Specific SOTR-drug Interactions
Monoclonal antibodies						
Bamlanivimab	Not authorized	Recombinant neutralizing human IgG1 monoclonal antibody that binds to spike protein of SARS-CoV-2	Alpha	Treatment of mild to moderate high-risk COVID-19 patients (12 years of age and older weighing at least 40 kg) • 700 mg IV once as a single IV infusion	Insufficient/no data in pregnancy, lactating patients, and pediatric patients	None
Bamlanivimab-etesevimab	Not authorized	Recombinant neutralizing human IgG1 monoclonal antibodies that binds to different overlapping epitopes of spike protein of SARS-CoV-2	Alpha Delta	Treatment of mild to moderate high-risk COVID-19 patients • 700–1400 mg IV once as a single IV infusion Post-exposure prophylaxis • 700–1400 mg IV once as a single IV infusion	Insufficient/no data in pregnancy, lactating patients, and pediatric patients	None
Bebtelovimab	Authorized under emergency use authorization	Recombinant neutralizing human IgG1 monoclonal antibody that binds to spike protein of SARS-CoV-2	Alpha Beta Gamma Delta Omicron	Treatment of mild to moderate high-risk COVID-19 patients (12 years of age and older weighing at least 40 kg) • 175 mg administered as a single IV injection over at least 30 s	No contraindications identified based on the limited available data available	None
Casirivimab-imdevimab	Not authorized	Recombinant neutralizing human IgG1 monoclonal antibodies that binds to non-overlapping epitopes of spike protein of SARS-CoV-2	Alpha Beta Gamma Delta	Treatment of mild to moderate high-risk COVID-19 patients (12 years of age and older weighing at least 40 kg) • 600–600 mg once as a single infusion administered together as a single IV infusion or by subcutaneous injection Post-exposure prophylaxis (12 years of age and older weighing at least 40 kg) • 600–600 mg once as a single infusion administered together as a single IV infusion or by subcutaneous injection	Insufficient/no data in pregnancy, lactating patients, and pediatric patients	None
Sotrovimab	Authorized under emergency use authorization	Recombinant neutralizing human IgG1 monoclonal antibody that binds to spike protein of SARS-CoV-2	Alpha Beta Gamma Delta Omicron	Treatment of mild to moderate high-risk COVID-19 patients (12 years of age and older weighing at least 40 kg) • 500 mg IV once as a single IV infusion	Insufficient/no data in pregnancy, lactating patients, and pediatric patients	None
Tixagevimab-cilgavimab	Authorized under emergency use authorization	Recombinant neutralizing human IgG1 monoclonal antibodies that binds to non-overlapping epitopes of spike protein of SARS-CoV-2	Alpha Beta Gamma Delta Omicron	Pre-exposure prophylaxis (12 years of age and older weighing at least 40 kg) • 150–150 mg as two separate IM injections	Insufficient/no data in pregnancy, lactating patients, and pediatric patients	None
Antivirals						
Molnupiravir	Authorized under emergency use authorization	Incorporates into SARS-CoV-2 RNA resulting in accumulation of errors in the viral genome leading to replication inhibition	Alpha Beta Gamma Delta Omicron	Treatment of mild to moderate high-risk COVID-19 patients (18 years of age and older) • 800 mg PO every 12 for 5 days	-Embryo-fetal toxicity: not recommended during pregnancy -Bone and cartilage toxicity: not authorized in patients less than 18 years of age -Use reliable contraception during treatment and after last dose (4 days after for females, 3 months for males) -Has not been evaluated in transplant recipients and may promote mutations in the spike protein of SARS-CoV-2	None
Nirmatrelvir-ritonavir	Authorized under emergency use authorization	Peptidomimetic inhibitor of the SARS-CoV-2 main protease/3C-like protease/nsp5 protease leading to replication inhibition	Alpha Beta Gamma Delta Omicron	Treatment of mild to moderate high-risk COVID-19 patients (12 years of age and older weighing at least 40 kg) • 300–100 mg PO twice daily for 5 days • Dosage adjustment needed when eGFR < 60 ml/min • Not recommended in severe renal impairment (eGFR < 30 ml/min) or severe hepatic impairment (Child–Pugh Class C)	Significant drug-drug interactions. Not recommend in patients who also take drugs highly dependent on CYP3A for clearance	-Ritonavir will increase levels of drugs metabolized by P450 CYP3A and increase levels of sirolimus, everolimus, tacrolimus, and cyclosporine -Will also interact with antifungal therapy such as azoles
Remdesivir	FDA-approved	Inhibitor of the SARS-CoV-2 RNA-dependent RNA polymerase leading to replication inhibition	Alpha Beta Gamma Delta Omicron	Treatment for hospitalized COVID-19 patients (12 years of age and older weighing at least 40 kg) • 200 mg IV once, followed by 100 mg daily—up to 10 days total Treatment for non-hospitalized mild to moderate high-risk COVID-19 patients (12 years of age and older weighing at least 40 kg) • 200 mg IV once, followed by 100 mg daily—up to 3 days total Do not administer if eGFR less than 30 ml/min Consider discontinuing if ALT levels increase to greater than 10 times the upper limit of normal or ALT elevation of signs/symptoms of liver inflammation	Insufficient data in pregnancy and lactating patients	None
Immunomodulators						
Tocilizumab	Authorized under emergency use authorization	Binds to IL-6 receptors which results in inhibition of IL-6 mediated signaling	n/a	Treatment for hospitalized COVID-19 patients (2 years of age and older) who are receiving systemic corticosteroids and requires supplementation oxygen or on ECMO • 30 kg and greater—8 mg/kg IV as a single infusion • Less than 30 kg—12 mg/kg as a single infusion • One additional infusion may be administered at least 8 h after initial infusion • Not recommended if ALT/AST above 10 times upper limit of normal range	Do not administer during any other concurrent active infection	None
Sarilumab	Not authorized	Binds to IL-6 receptors which results in inhibition of IL-6 mediated signaling	n/a	Alternative to tocilizumab • 400 mg IV once as a single infusion • Not recommended if ALT above 5 times upper limit of normal	Do not administer during any other concurrent active infection	None
Dexamethasone	Not authorized	Glucocorticoid that decreases inflammation by suppression of neutrophil migration	n/a	Hospitalized COVID-19 patients requiring supplemental oxygen or ECMO • 6 mg PO daily for up to 10 days or until hospital discharge		None
Baricitinib	Authorized under emergency use authorization	Janus kinase inhibitor	n/a	Treatment for hospitalized COVID-19 patients (2 years and older) who require supplementation oxygen or on ECMO • 9 years and older—4 mg once daily • 2 years to less than 9 years of age—2 mg once daily • Duration for 14 days or until hospital discharge (whichever is earlier)	Embryo-fetal toxicities have been observed in animals dosed in excess of human exposure	None

*As of February 2022.

### Oral Antivirals

As of the end of 2021, the FDA granted EUAs for two different oral antivirals, nirmatrelvir/ritonavir (Paxlovid, Pfizer), and molnupiravir (Lagevrio, Merck) [28, 29]. Due to limited options, the appeal of these medications is for the use in the outpatient setting. Paxlovid, as a potent inhibitor of CYP3A, will have significant drug-drug interactions with calcineurin inhibitors, mTOR inhibitors, and azole antifungals, which will significantly limit its use in SOTR [30]. Additionally, molnupiravir will likely require extremely high doses to achieve effect without increasing viral resistance [31]. Therefore, the American Society of Transplantation (AST) currently does not recommend use of either of these medications as first-line agents to treat mild COVID-19 in SOTR [32]. See Table 1 for details on for Paxlovid and molnupiravir.

### Corticosteroids

Corticosteroids were not initially recommended in the treatment of SARS-CoV-2 due to the theoretical concern regarding delayed viral clearance [33]. However, the RECOVERY Trial, a controlled, open-label trial that randomized over 2000 patients to receive steroids, demonstrated hospitalized patients with COVID had significantly lower 28-day mortality with 6 mg of dexamethasone for up to 10 days [34••]. This finding was borne out in participants requiring supplemental oxygen only. The trial did not provide information on SOTR [34••]. The CoDEX trial of dexamethasone also displayed more ventilator-free days than placebo in patients with moderate to severe acute respiratory distress syndrome (ARDS) [35•]. These studies are in contrast to the CAPE COVID study, which was stopped once RECOVERY results were available, that demonstrated no difference in mortality among patients on low-dose hydrocortisone versus placebo [36•]. This trial included less than 10 patients with immunosuppressive conditions or on immunosuppression and evaluated hydrocortisone, rather than dexamethasone [36•]. This decreased mortality rate in critically ill patients with COVID-19 was also noted in a WHO meta-analysis of these numerous RCTs [37, 38].

Given these findings, the type of steroid and dose of steroids have been examined in a few large trials. Hydrocortisone did not reduce treatment failure at day 21; however, this study was terminated early, leading to an underpowered study [36•]. Likewise, the REMAP-CAP randomized open-label trial of hydrocortisone did not increase support-free days, regardless of the dose of hydrocortisone [39]. Dexamethasone dosing was studied in the COVID STEROID 2 trial, which was a multinational blinded randomized controlled trial of dexamethasone 12 mg compared to 6 mg in adults with severe hypoxemia due to COVID-19. This study found that there was no difference in life support-free days when comparing the doses, though the duration was variable in the treatment group [40•].

SOTRs were not specifically evaluated in sub-group analyses of these trials. Early case studies of transplant recipients noted that patients were already on low-dose steroids for immunosuppression and had similar outcomes of COVID-19 [41]. As the pandemic has progressed, extensive use of steroids in hypoxic and critically ill patients has proven beneficial to decrease hyperinflammation related to COVID-19 [2•, 42]. Risk of secondary opportunistic infections in already immunosuppressed patients is an ongoing concern. In our experience, SOTRs who are hypoxic or crucially ill may still benefit from the use of dexamethasone. In SOTR already on a steroid, it is likely safe to continue.

### Immunomodulators

IL-6 inhibitors, specifically tocilizumab and sarilumab, have been examined in large, randomized controlled trials and have exhibited a beneficial effect among patients with progressive moderate to severe COVID-19 who are not yet on mechanical ventilation [43•, 46]. The RECOVERY trial compared tocilizumab and standard of care arms in hospitalized patients with oxygen saturation (SpO2) less than 92% and CRP equal to or greater than 75 mg/L. The tocilizumab arm was associated with reduced all-cause mortality in this specific population [47••]. Both tocilizumab and sarilumab were evaluated in the REMAP-CAP trial, and both treatment arms had higher rate of in-hospital survival and shorter duration of organ support than standard of care. This effect was strongest in those with the highest CRP and enrolled patients within 24 h of ICU admission [48, 49]. While both RECOVERY and REMAP trials confirmed benefit of both tocilizumab and sarilumab among the sickest patients with COVID-19, it is important to note that patients were receiving dexamethasone concomitantly in these trials. Additionally, other studies of tocilizumab have not been as overwhelmingly positive. The COVACTA double-blind RCT of tocilizumab in hospitalized patients showed no survival benefit; however, the median times for recovery and ICU length of stay was shorter in the tocilizumab group than placebo [43]. The EMPACTA double-blind RCT of tocilizumab determined that it lowered rates of mechanical ventilation, ECMO, or death by day 28 but provided no benefit for all-cause mortality at that time [45].

Despite these trials demonstrating advantages of tocilizumab, the REMDACTA double-blind RCT of tocilizumab and remdesivir in hospitalized patients with severe COVID-19 pneumonia found that the combination of remdesivir plus tocilizumab did not shorten time to discharge and there was no difference in mortality between arms [50]. Nevertheless, tocilizumab was given FDA EUA in June of 2021 [51].

In STOR, use of tocilizumab has been evaluated in observational studies, calling into question the safety and efficacy of additional immunosuppression in this population [52]. In an observational cohort of 80 kidney transplant recipients with severe COVID-19 by Perez-Saez et al., in patients who received tocilizumab, the mortality rate was noted to be 32.5%, significantly higher than early studies that reported mortality in SOTR up to 28% [21, 52, 53]. This finding correlated with higher inflammatory markers at baseline in the treated SOTR [52]. Additionally, in a matched cohort study of 117 SOTR, 29 patients received tocilizumab compared to matched controls and no benefit in mortality was found [54]. This study noted that secondary infections occurred frequently in the tocilizumab treatment group, 34% versus 24% in the control group, but it was not statistically significant. The study concluded that tocilizumab is safe to use in SOTR with minimal impact on the net state of immunosuppression [54]. Tocilizumab may be beneficial in SOTR with severe COVID-19 and elevated inflammatory markers.

### Kinase inhibitors

Of the kinase inhibitors currently in use, there are randomized clinical data only for baricitinib and ruxolitinib in COVID-19. The ACTT-2 trial randomized over 1000 hospitalized patients to receive remdesivir plus baricitinib or remdesivir plus placebo [55••]. The time to recovery was significantly reduced with combination treatment, predominantly in the patients on high-flow oxygen or noninvasive ventilation. Secondary outcomes showed combination therapy was associated with better clinical status at day 15 in this same subgroup [55••]. Patients who were already getting corticosteroids were excluded from this trial, minimizing confounding. Similar observations were found in the COV-BARRIER study, which compared baricitinib alone with placebo. However, nearly 80% of patients in this trial were on concomitant dexamethasone [56••]. Based on these data, the NIH recommends either baricitinib or tocilizumab in combination with dexamethasone or dexamethasone plus remdesivir for hospitalized patients with rapidly worsening oxygen requirement on high-flow or noninvasive ventilation and high inflammatory markers [7••]. The FDA EUA for baricitinib expands the use to include patients who are mechanically ventilated or on ECMO [57]. Ruxolitinib, an alternate kinase inhibitor, was not associated with faster clinical improvement [58].

Data on baricitinib in SOTR is sparse. Bodro et al. discuss use of baricitinib in 33 kidney transplant recipients (KTR) admitted to the hospital in the early stages of the pandemic [59]. This study found that KTR had better subjective outcomes when treated with anticytokine therapy. Despite the lack of specific studies in SOTR, the NIH still recommends use of baricitinib for patients with severe or critical COVID-19 disease with rapid progression [60]. While Marconi et al. did not find a statistically significant difference in rates of adverse effects, including secondary infection, in the treatment versus control arms, monitoring for secondary infections remains a key concern for patients already on immunosuppression [56••]. The American Society for Transplantation concludes that baricitinib is a useful treatment for severe COVID-19 in patients who progress despite remdesivir and dexamethasone and advises that SOTR who receive baricitinib should be monitored closely for secondary infections [42]. In our experience, baricitinib has been well tolerated when used in patients without proven secondary infection.

### Monoclonal Antibodies (MABs)

In randomized controlled trials of the various monoclonal antibodies, outcomes were not stratified by organ transplant recipients specifically. Yet all of them were able to demonstrate improved outcomes among patients with risk factors for more severe COVID-19 disease and hospitalization, namely, reducing hospitalization rates [61].

Bamlanivimab was studied in the Blocking Viral Attachment and Cell Entry with SARS-CoV-2 Neutralizing Antibodies (BLAZE-1) trials, double-blind RCT with three phases. The first phase compared bamlanivimab versus placebo, with a post hoc analysis of high-risk patients revealing lower hospitalizations in the study drug arm [62]. Phase 2, however, did not demonstrate a difference between placebo and bamlanivimab alone, only the combination of bamlanivimab plus etesevimab resulted in decreased viral load. All study arms had fewer hospitalizations [63]. Studies in SOT of both bamlanivimab alone and bamlanivimab plus etesevimab showed that these monoclonal antibodies were safely used and helped prevent COVID-19 disease [64–66]. The EUA for bamlanivimab plus etesevimab was granted on February 2021 for high-risk patients with mild to moderate COVID-19 [67, 68]. As of August 2021, this combination was no longer recommended due to decreased susceptibility of Delta and Omicron variants [7••].

Casirivimab/imdevimab (brand name Regeneron) was studied in a three-phase RCT. Regeneron significantly decreased viral level from day 1 through day 7 when compared to placebo [61]. This effect was most notable among seronegative patients, those with no more than 7 days of symptomatic disease. The FDA issued an EUA for Regeneron for mild to moderate COVID-19 at high risk of progression in February 2021 [69]. In a study of 12 kidney transplant recipients, in patients with mild COVID-19 disease, casirivimab/imdevimab resulted in rapid resolution of symptoms and no one required repeat hospitalization [70]. With the latest Omicron variant wave, Regeneron is no longer recommended as it is ineffective against this variant [7••, 71].

Sotrovimab is currently being used against Omicron variant. Sotrovimab targets a portion of the receptor binding domain, which remains highly conserved in the Omicron variant [72]. Ongoing data from phase 3 trial shows lower hospitalization rates in outpatients given sotrovimab versus placebo [73]. Gupta et al. conducted a double-blind placebo controlled trial of sotrovimab that demonstrated an 85% reduction in hospitalization or death in patients who received sotrovimab compared to placebo [72]. Severely immunocompromised patients were excluded from this study [74]. However, we have used sotrovimab in SOTR during the latest Omicron surge with anecdotal success. As of February 2022, FDA EUA for sotrovimab remains active [75].

Bebtelovimab is the most recent monoclonal antibody available to treat COVID-19 and works by binding to the COVID-19 spike protein similar to other monoclonals. To date, bebtelovimab seems to retain activity against both the omicron variant and the BA.2 omicron subvariant [76, 77].

### Vaccination and Prophylaxis

Vaccination against COVID-19 has helped limit the spread of the disease. Available vaccines in the USA include the mRNA vaccines, mRNA-1273 (Moderna) and BNT162b2 (Pfizer), and Ad26.COV2.S (J&J). Recommendations for SOTR are to prioritize the mRNA vaccine, as they appear to mount a stronger humoral response compared to adenovirus vector vaccines, such as the J&J vaccine [5••]. Current recommendations regarding vaccine schedules include three doses in the primary series of mRNA vaccines and a booster 3 months after [5••]. If the patient received Ad26.COV2.S (J&J), it is recommended they receive a booster with a mRNA COVID-19 vaccine at least 2 months after the 2nd dose [78].

SOTR response to vaccination has been widely researched and variable in the literature. Antibody response in SOTR after receiving COVID-19 has evaluated spike protein IgG or receptor-binding domain IgG [79•, 80, 81, 82, 83]. These studies have looked at rates of detectable antibodies after two doses of mRNA vaccines, and response is extremely variable, ranging from 0 to 64% [79•, 84, 85]. Improved rates of antibody response have been noted with addition of third dose of mRNA vaccine [86]. T cell response ranges as well, but can be present despite absent antibody [84, 87, 88]. A few studies have gauged clinical effectiveness of the vaccines and show reduced severity of disease in symptomatic COVID-19 and reduced mortality [83, 89, 90]. One single-center study noted an almost 80% reduction in risk of symptomatic COVID-19 in vaccinated SOTR [90]. Notably, these studies were performed prior to the Omicron variant surge.

Pre-exposure prophylaxis is now a consideration with the arrival of tixagevimab + cilgavimab (Evusheld) [91]. Evusheld is indicated in adult and pediatric patients older than 12 years old who are not currently infected with COVID-19 nor have recent exposure to COVID-19 [92]. In the PROVENT trial, Evusheld was shown to decrease the likelihood of developing symptomatic COVID-19 by 77% in a 6-month follow-up period [93•]. Further data from the TACKLE study indicates that Evusheld may be used as prevention and possibly treatment in the future [94]. Patients considered for Evusheld should be moderately to severely immunocompromised and unlikely to mount an immune response to vaccination. Alternatively, recipients should have a contraindication to vaccination due to prior adverse reaction to mRNA vaccine. While this option provides recent SOTR with protection against COVID-19, AST still recommends prioritizing vaccination due to scarcity of Evusheld as it is distributed [42, 95].

### Disproven and Not Recommended Medications

Notably, certain medications have been investigated as preventive measures from contracting COVID-19. Ivermectin, an anti-parasitic medication, inhibits replication of viruses in vitro. Meta-analysis of numerous trials does not show any benefit to ivermectin [96, 97].

**Fig. 1:**
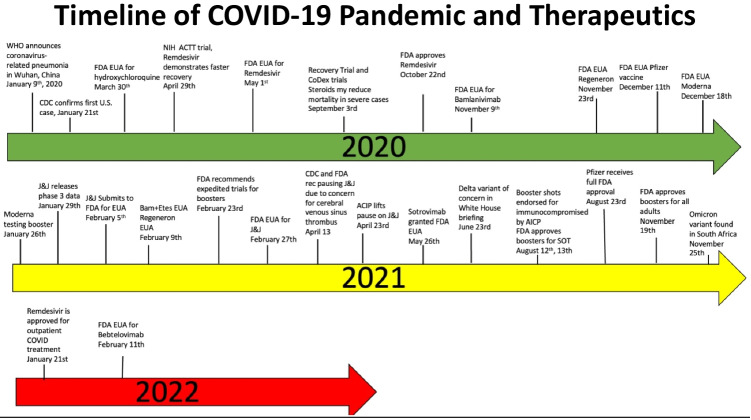
Timeline of COVID-19 pandemic and therapeutics

Data on the use of chloroquine and hydroxychloroquine have not been shown to benefit COVID-19. The FDA EUA was revoked for both drugs due to ineffectiveness, and both have potential to prolong QTc and cause harm (see Fig. 1) [7••, 98].

Colchicine has also been investigated as a potential treatment for hyperinflammatory syndrome that accompanies COVID-19. No clinical benefit has been demonstrated, and colchicine is not currently recommended [42, 99].

Fluvoxamine is a selective serotonin reuptake inhibitor (SSRI) that may reduce cytokine production as demonstrated in mouse models [100]. In the STOP COVID trial, a double-blind RCT of non-hospitalized patients, the fluvoxamine treatment arm did have 0% clinical deterioration compared to 8.3% in placebo arm [101]. However, this study had a short follow-up period with a significant number of patients stopping responding prior to 15-day follow-up [42]. The TOGETHER trial also studied fluvoxamine in a RCT, but statistically showed no difference in primary outcome [102]. At this time, neither the NIH nor the IDSA guidelines recommend routine use of fluvoxamine in the treatment of COVID-19 [7••, 98].

## Conclusion

COVID-19 therapeutics have gone through several iterations since the beginning of the pandemic in 2020. SOTRs have often been one of the populations most affected by this disease, and evidence on specific treatment in this population is limited. In our clinical practice, remdesivir remains the antiviral of choice for those hospitalized with moderate COVID-19. Additional treatments for moderate to severe disease include corticosteroids, tocilizumab, or baricitinib. Outpatient treatment for mild COVID-19 includes shorter courses of remdesivir, or oral antivirals, or monoclonal antibodies. As new therapeutics evolve for this virus, it is imperative that immunocompromised patients, specifically SOTRs, are included in studies. Providers should continue ongoing vaccination campaigns for those who remain unvaccinated or partially vaccinated. Prevention with pre-exposure prophylactic medications could provide protection for many who cannot mount an immune response to the vaccine. The progress in the treatment of COVID-19 should include SOTR as the medical community navigates the future of this pandemic.
